# A Case of Fournier’s Gangrene in a Patient With Malignant Priapism

**DOI:** 10.7759/cureus.58465

**Published:** 2024-04-17

**Authors:** Sotirios Kapsalos, Stamatios Katsimperis, Themistoklis C Bellos, Panagiotis Angelopoulos, Panagiotis Neofitou, Panagiotis Deligiannis, Titos Markopoulos

**Affiliations:** 1 Urology, Second Department of Urology, National and Kapodistrian University of Athens, Sismanogleio General Hospital, Athens, GRC; 2 Urology, Second Department of Urology, National and Kapodistrian University of Athens, Sismanogleio General Hospital, Athens, GRC, Athens, GRC

**Keywords:** penectomy, total penectomy, malignant priapism, priapism, fournier's gangrene

## Abstract

Fournier’s gangrene is a rare and potentially life-threatening type of necrotizing fasciitis that affects the genital and perineal regions. Malignant priapism is a rare and serious medical condition characterized by persistent and painful erection of the penis that is not associated with sexual arousal or stimulation. We present a case of a 77-year-old man with concurrent Fournier’s gangrene and malignant priapism. He first underwent surgical debridement to remove necrotic tissue and aspiration of blood from the corpora cavernosa. Then a palliative penectomy was performed. The patient succumbed to severe sepsis and died after 14 days of hospitalization.

## Introduction

Fournier’s gangrene is a rare condition with an overall incidence of 1.6 cases per 100,000 males per year [1,2]. The disease usually appears in immunocompromised patients with diabetes, obesity, and malignant neoplasms. The basic treatment includes emergency surgical intervention and antibiotic therapy. Malignant priapism is a rare type of priapism, often associated with hematologic disorders such as sickle cell disease, leukemia, or metastatic malignancies [3,4]. Here, we report a case of a patient with concurrent Fournier gangrene and malignant priapism who presented to the emergency department with swelling, redness, and pain in the genital and perineal areas and a painful erection.

## Case presentation

**Figure 1:**
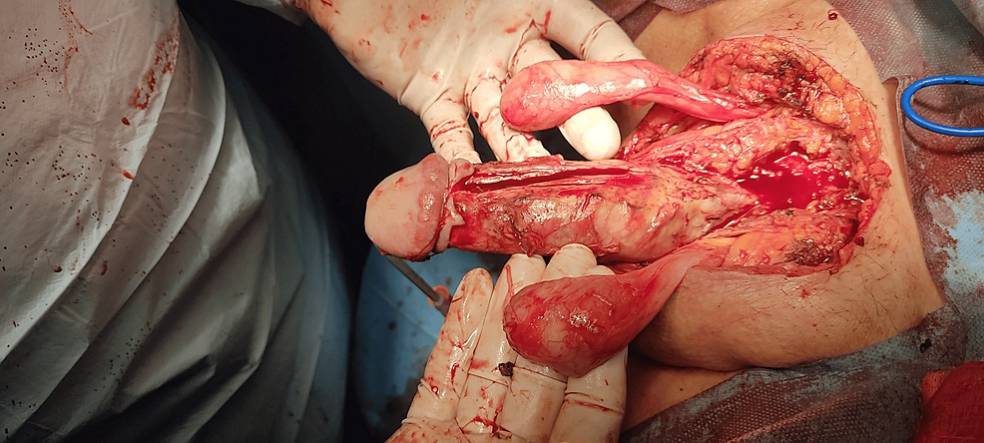
Surgical debridement of necrotic tissue and extensive exploration of perineal and genital regions

**Figure 2:**
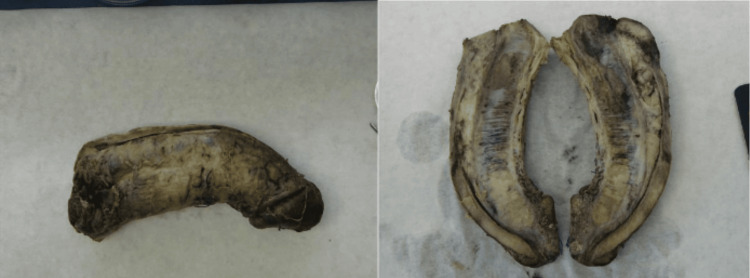
Gross pathology specimen

A 77-year-old man presented to the emergency department complaining of swelling and pain in the genital area and an unintentional erection for 72 hours. From his medical history, he had metastatic colorectal cancer with hepatic and lung metastases. The patient was under palliative chemotherapy. The clinical examination revealed severe inflammation of the perineal and genital areas with tenderness and foul-smelling discharge. The presence of priapism was also visible. The lab investigations showed an increase in white blood cells and C-reactive protein. Blood and urine cultures were taken. We started antibiotic therapy with piperacillin, tazobactam, and vancomycin intravenous. Computed tomography confirmed extensive soft tissue necrosis in the perineal and genital regions. The patient underwent aggressive surgical debridement of necrotic tissue, and an extensive exploration of the perineal and genital regions was performed (Figure 1). Irrigation of corpora cavernosa was unsuccessful, and a penile shunt was performed to relieve priapism. The patient was transferred for 24 hours to the intensive care unit. He continued his treatment at the urology clinic. Despite the aggressive management, the patient’s priapism had no improvement, and his clinical condition was getting worse. For this reason, a palliative penectomy was performed. The patient continued his treatment in the intensive care unit. After two days, the patient died due to severe sepsis. The histological examination of the penis revealed infiltration of corpora cavernosa from malignant cells of adenocarcinoma of the colon (Figure 2).

## Discussion

Concurrent presentation of Fournier's gangrene and malignant priapism is an extremely rare but potentially life-threatening condition. Fournier's gangrene is a polymicrobial necrotizing fasciitis affecting the perineal and genital regions, often precipitated by local trauma or underlying comorbidities such as diabetes mellitus [5]. It is crucial to immediately recognize a patient with suspected Fournier gangrene [6,7]. Broad-spectrum antibiotics must be administered in the very initial hours. Surgical debridement of necrotic tissue is the most determinant factor in the prognosis of the disease. The mortality rate is 20-30% for the patients who do receive treatment on time [8]. Malignant priapism, characterized by persistent, non-ischemic penile erection, is a rare complication of advanced cancer associated with a poor prognosis [9]. The true incidence of malignant priapism remains unknown due to the rarity of the disease and the lack of information regarding this clinical entity. The most common malignancies that lead to penile metastases and malignant priapism are urogenital tumors, accounting for up to 70% of all cases [10]. The second most common malignancy is tumors of the gastrointestinal tract [11]. Concurrent Fournier’s gangrene and malignant priapism is a very rare entity that is poorly studied, with no cases reported. The existence of priapism increases the morbidity of Fournier’s gangrene. Our patient presented to the emergency department with severe sepsis in a very bad general condition. He had a known history of metastatic cancer, and he was under palliative chemotherapy. All these factors are related to a poor prognosis.

## Conclusions

Fournier’s gangrene is a rare disease with high mortality, even if the appropriate treatment is given. The existence of malignant priapism and concurrent Fournier’s gangrene is extremely rare and predisposes to a very poor prognosis. To the best of our knowledge, this is the first report of such a rare clinical case. We tried to highlight the aggressive treatment needed when dealing with such patients and the poor outcomes that usually follow.
